# The Clinical Accuracy and Risk Stratification in End of Therapy ^18^F-FDG PET/CT in Burkitt Lymphoma

**DOI:** 10.3389/fonc.2021.625436

**Published:** 2021-04-21

**Authors:** Yi Wen Mo, Zi Zheng Xiao, Yuan Wei, Xin Ling Li, Xu Zhang, Wei Fan

**Affiliations:** Sun Yat-sen University Cancer Center (SYSUCC), Guangzhou, China

**Keywords:** Deauville 5-point scale, Burkitt lymphoma, prognosis, FDG, PET/CT

## Abstract

**Purpose:**

Burkitt lymphoma (BL) is an invasive lymphoma subtype with FDG avid at ^18^F-FDG PET/CT, but there is currently no validated criterion in treatment evaluation and prognosis prediction. The aim of this study was to analyze the clinical accuracy of ^18^F-FDG PET/CT in Burkitt lymphoma in end of therapy PET/CT (EOT-PET) to assess the treatment response in BL and conduct a survival analysis with different Deauville 5-point score (DS) cutoff values.

**Materials and Methods:**

A total of 189 patients were retrospectively included: 97 underwent baseline PET/CT and all underwent EOT-PET. Survival curves were plotted according to the Kaplan-Meier method. Different DS cutoff values in EOT-PET were evaluated for risk stratification in Burkitt lymphoma.

**Results:**

The median progression free survival (PFS) and overall survival (OS) were 52 and 53 months, respectively. Applying the conventional DS 4 to 5, there was significant difference in outcome between EOT-PET negative and positive patients. However, the positive predictive value (PPV) (28.3% for PFS and 26.4% for OS) is low despite a high negative predictive value (NPV) (94.1% for OS and 94.9% for OS). When we moved the cutoff point to DS 5, the PPV was improved evidently (88.2% for PFS and 82.3% for OS) with the satisfactory NPV simultaneously (95.3% for PFS and 95.9% for OS).

**Conclusions:**

EOT-PET results using DS significantly related with PFS and OS. DS of 5 may be a better cutoff point at the end of treatment to determine whether patients have a significant risk of recurrence or progress.

## Background

Burkitt lymphoma (BL) derived from germinal center B cells, which is a highly aggressive B cell non-Hodgkin lymphoma (1). It has an intimate relationship with Epstein-Barr virus (EBV) infection (2, 3) and is divided into three main forms: endemic, sporadic, and immunodeficiency-associated variants (4, 5). Burkitt’s lymphoma is extremely sensitive to chemotherapy and is potentially curable with short and intensive treatment programs, with survival ranges from over 80 to 50% related to the stage and age of diagnosis (6, 7).

The Conventional staging work rely on computerized tomography (CT) imaging, blood tests, bone marrow aspiration and biopsy, cerebrospinal fluid (CSF) (8). Fluorine-18-fluorodeoxyglucose positron emission tomography/CT (^18^F-FDG PET/CT) combines a PET scanner detector with a helical CT multidetector. It offers more sensitive and specific imaging than either modality alone (9). Although several promising studies (10–15) had focused on the role of ^18^F-FDG PET/CT in BL, interpretation has not been standardized until now.

According to the updated international guidelines, 18F-FDG PET/CT has been recommended as the gold standard in staging and response assessment at the end of treatment for FDG avid lymphomas, such as Hodgkin lymphoma (HL) and diffuse large B-cell lymphoma (DLBCL) (16, 17). Deauville 5-point scale (DS), which based on visual analysis, as metabolic response assessment criterion (17), is a continuous parameter reflecting the dynamic response to the treatment. DS1-3 was defined as a negative result, and DS4-5 was defined as a positive result (17). Considering almost 100% cases of BL are FDG-avid (17), the DS response assessment scale can be applied to the patients with BL. However, this method has only initial and controversial evidences in BL, and the potential effect has not been confirmed (13, 15). In addition, these studies have difficulty in obtaining reliable results because of their small sample size.

The purposes of our study, performed in BL patients who underwent end of therapy PET/CT (EOT-PET), were (1) to analyze the clinical accuracy of EOT-PET in BL; and (2) to conduct a survival analysis with different DS cutoff values for further refinement the response assessment from a different perspective in a large group.

## Patients and Methods

### Patients

376 patients were retrospectively identified with histological proven BL in our cancer center between January 2010 and December 2018. Patients with concomitant malignancy and without EOT-PET were excluded. 189 patients were recruited finally. 97 of the 189 patients underwent baseline PET/CT. Patients were classified at the time of diagnosis by the Ann Arbor system. The medical records of these patients were reviewed and analyzed. We collected the age, gender, immune system condition, nodal or extranodal localization, B symptom, Eastern Cooperative Oncology Group (ECOG) performance status, International prognostic index (IPI) score, lactated hydrogenase (LDH) level, treatment modality and follow-up data. The cutoff values of IPI score, ECOG performance status and LDH level are 2, 2 and 245 U/L, respectively. According to Ann Arbor classification, tumor stage is divided in early (I and II) and advanced (III and IV) stage. This retrospective study was approved by the ethics committees of Sun Yat-sen University Cancer Center. Informed patient consent was not required.

### 18F-FDG PET/CT Scan Protocol

All patients fasted for six hours prior to ^18^F-FDG administration. Patients with a blood glucose level of 200 mg/dL (11.1 mmol/L) or higher at the time of injection were rescheduled. 18F-FDG PET/CT scans were performed using integrated PET/CT scanners (Discovery ST, GE Healthcare, Waukesha, Wis, USA; or Biograph mCT, Siemens Healthcare, Henkestr, Germany). Patients were injected with 5.55 ± 0.74 MBq (0.15 ± 0.02 mCi) with Discovery ST and 3.7 ± 0.37 MBq (0.1 ± 0.01 mCi) with Biograph mCT, per kilogram of body weight. Imaging was performed 50 to 80 minutes after the injection of FDG. The scanning range was form the skull to the mid-thigh in an arm-up position. The low dose CT scan was obtained prior to the PET scan for attenuation correction using the following parameters: automatic tube current modulation, tube voltage 140 kV, collimation 16 × 1.25 mm, rotation time 0.8 s, slice thickness 3.75 mm for the Discovery ST or tube current 80–200 mAs, voltage 120 kV, collimation 32 × 1.25 mm, rotation time 0.5 s, slice thickness 3 mm for the Biograph mCT.

The subsequent emission images were obtained with three minutes per bed position and two-dimensional (2D) in Discovery ST or with 1.5-2 minutes per bed position and three-dimensional (3D) in Biograph mCT with six to eight bed positions. PET images were reconstructed using the ordered subsets expectation maximization iterative image reconstruction method with a slice thickness of 3.25 mm (2D) in a 128 × 128 matrix or with 2 mm (3D) in a 200 × 200 matrix.

### Image Analysis

The ^18^F-FDG PET/CT data obtained at the end of treatment were blindly and independently reviewed by two nuclear medicine physicians with over five years of experience with PET/CT in lymphoma imaging by visual evaluation. The quantitative method is used to determine the score when there is disagreement in visual evaluation. We also consulted a more experienced nuclear medicine physician in lymphoma imaging if opinions are not uniform. The EOT-PET was obtained after the completion of chemotherapy at least 3 weeks.

The PET/CT results were classified using 5-DS (17): 1 = no uptake, 2 = uptake less than mediastinum, 3 = uptake between mediastinum and liver, 4 = uptake moderately more than liver, 5 = uptake markedly higher than liver or new sites of disease. During analysis, uptake with a score of 5 was considered to be 200% of the liver maximum standard uptake value (SUVmax). DS 4 and 5 were considered a single positive category like previous studies (18, 19), whereas DS 1 to 3 were considered as a single negative category.

### Statistical Analysis

Treatment response was evaluated according to the International Working Group Recommendations for Response Criteria for non-Hodgkin’s lymphomas (17). Progression free survival (PFS) was calculated from the start of the treatment to the date of first disease progression, relapse, or the date of last follow-up. Overall survival (OS) was calculated from the start of the treatment to the date of death from any cause or to the date of last follow-up. The results of laboratory examinations, clinical data, and conventional diagnostic procedures such as ultrasonography, CT, and biopsy of suspicious residual disease (if available) decided the disease state.

Univariate and multivariate Cox proportional hazards regression model were used to assess the relation between potential variables and PFS or OS. Survival curves were plotted according to the Kaplan–Meier method and differences between groups were analyzed with a two-tailed log rank test. The sensitivity, specificity, negative predictive values (NPV) and positive predictive values (PPV) were calculated via the standard definition (20). To evaluate the diagnostic power, we calculated the area under the curve (AUC) on the basis of receiver operating characteristic (ROC). All statistical analysis was performed with Statistical Package for Social Science (SPSS) version 20.0 (IBM, Chicago, IL, USA). P-values less than 0.05 were regarded as statistically significant.

## Results

### Patients Characteristics and Outcomes

**Figure 1:**
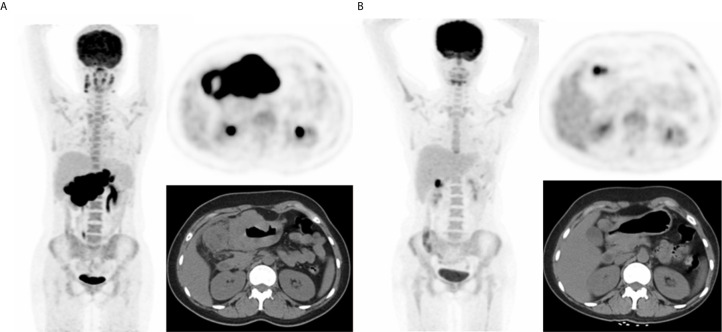
A representative case of a 25-year-old female with stage IIIA BL and both nodal and extranodal disease. Baseline maximum intensity projection (MIP) **(A)** showing hypermetabolic lesion in stomach and abdominal nodes. End of treatment PET/CT **(B)** showing high 18F-FDG uptake residual lesion in the lymph node of gastric, considering a partial response compared to baseline scan. This patient died nine months after baseline PET/CT.

**Figure 2:**
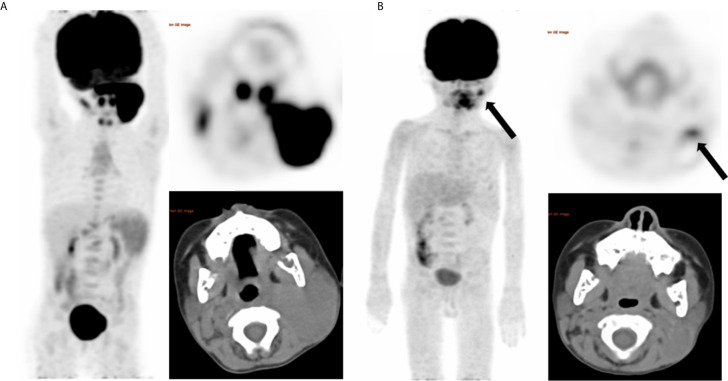
A representative case of a 4-year-old female with stage IIIB BL and nodal disease. Baseline maximum intensity projection (MIP) **(A)** showing hypermetabolic lesion in cervical nodes. End of treatment PET/CT **(B)** showing moderate 18F-FDG uptake residual lesion in the cervical node (SUVmax = 4.1), considering Deauville score of 4 and a partial response. And the residual lesion dissection was performed, proving no viable lymphoma cell. After a follow up time of 62 months, this patient did not develop relapse and was alive.

Among 189 patients with histologically proven BL, 141(75%) were male and 48(25%) were female. The average age was 19 years (range, 1 to 75 years). Bulky disease was present in 29% and B symptoms in 36.5% of patients; while extranodal BL involvement was identified in 140 patients (74%). According to the Ann Arbor system, we found stage I in 16, stage II in 37, stage III in 55 and stage IV in 81 patients. 130 patients’ serum LDH levels were abnormal and 106 cases’ IPI scores were superior or equal to 2. The baseline clinical features of patients are summarized in **Table 1**. Based on the stage of disease, age, patient’s physical condition and institutional internal protocol, all patients were treated with chemotherapy regimen according to the institution’s standard protocol. The majority of children and adolescents (109, 57.7%) received intensive chemotherapy according to the B cell Non-Hodgkin’s Lymphoma (B-NHL) Berlin – Frankfurt - Mu¨nster (BFM) - 95 protocol or the B-NHL-BFM-90 protocol (21, 22). 27.5% of which underwent high-intensity chemotherapy, such as CODOXM/IVAC (cyclophosphamide, vincristine, doxorubicin, methotrexate, ifosfamide, etoposide, and cytarabine) and HyperCAVD (hyperfractionated cyclophosphamide, vincristine, doxorubicin, and dexamethasone), and 28 (14.8%) patients received moderate-intensity protocols such as CHOP (doxorubicin, vincristine, cyclophosphamide, and prednisone). A total of 150 patients (79.4%) received rituximab on the basis of routine chemotherapy and 25 patients (16.7%) showed DS 4 in this cohort. Of the other 39 patients who did not receive rituximab, 11 patients (28.2%) were DS 4. There were 41 patients (21.7%) receiving both surgery and chemotherapy. Only one patient underwent PET/CT scan four months after radiotherapy for response evaluation. 136 out of 189 enrolled patients (72.0%) achieved complete response (CR), 37 patients (19.5%) achieved partial response (PR) and16 patients (8.5%) showed progressive disease (PD) and only two of PD patients still survived up to the last follow-up. A total of 21 patients (11.1%) died at the last follow-up (**Figures 1** and **2**). For the entire cohort, median PFS was 52 months (range 3–122 months) and the median OS was 53 months (range 5–122 months).

**Table 1:** Patient Characteristics.

Parameters	N=189	%
Age(average-range)(years)	19	1-75
≤14	110	58
>14	79	42
Gender		
Male	141	75
Ann Arbor stage		
I	16	8
II	37	20
III	55	29
IV	81	43
B symptom		
Present	69	37
Extranodal disease	140	74
LDH level		
>245	130	69
ECOG performance status		
≥2	83	44
International prognostic index (IPI)		
Low	47	25
Low-intermediate	35	18
High-intermediate	90	48
High	17	9
Deauville 5-point score (DS)		
DS 1, 2, 3	136	72
DS 4	36	19
DS 5	17	8

ECOG, Eastern Cooperative Oncology Group; LDH, Serum lactate dehydrogenase.

### Cox Regression Models

Univariate and multivariate Cox regression models were performed to evaluate the prognostic significance of Clinical characteristics on PFS and OS, including age, gender, B symptom, LDH levels, Ann Arbor stage, ECOG performance status, extranodal disease, high IPI scores and EOT-PET results and summarized in **Table 2**. In univariate analysis, significant variables were LDH level (PFS: hazard ratio (HR) = 5.198, 95% confidence interval (CI) = 1.218 - 22.174, p = 0.012; OS: HR = 4.686, 95 CI% = 1.091 - 20.122, p = 0.038) and positive EOT-PET result (PFS: HR =1.056, 95 CI% = 1.028 – 1.084, p < 0.001; OS: HR = 1.057, 95 CI% = 1.027 – 1.087, p < 0.001) both for PFS and OS. After an adjustment for these covariates, LDH level (HR = 4.349, 95 CI% = 1.014 – 18.655, p = 0.048) and positive EOT-PET (HR =1.051, 95 CI% = 1.023 – 1.084, p < 0.001) were also the variables significantly associated with PFS and the positive EOT-PET was the only variable significantly associated with OS (HR =1.055, 95 CI% = 1.025 – 1.085, p < 0.001). There was no significant correlation among clinical scores on clinical features (age, gender, Ann Arbor stage, ECOG score, B symptom, extranodal disease, high IPI) and PFS or OS.

**Table 2:** Univariate and multivariate analyses for PFS and OS.

	Univariate analysis		Multivariate analysis		
	p value	HR (95%CI)	p value		HR (95%CI)
PFS					
age(>14 vs. ≤14)	0.134	1.970(0.816-4.754)			
Gender	0.809	0.875(0.321-2.383)			
Ann Arbor stage	0.226	1.989(0.644-6.150)			
ECOG score≥2	0.716	0.772(0.214-2.782)			
B symptom	0.841	1.136 (0.464-2.780)			
elevated LDH	0.012	5.198(1.218-22.174)	0.048		4.349 (1.014-18.655)
Extranodal disease	0.944	0.991(0.367-2.675)			
IPI score≥3	0.636	1.221(0.501-2.978)			
Positive EOT PET/CT (DS 4 or 5)	<0.001	1.056 (1.028-1.084)	<0.001		1.051 (1.023-1.080)
OS					
age(>14 vs. ≤14)	0.140	2.010(0.803-5.032)			
Gender	0.623	0.753(0.272-2.087)			
Ann Arbor stage	0.338	1.750(0.560-5.466)			
ECOG score≥2	0.846	0.870(0.240-3.161)			
B symptom	0.551	1.350 (0.538-3.388)			
elevated LDH	0.038	4.686(1.091-20.122)			
Extranodal disease	0.793	0.860(0.314-2.357)			
IPI score≥3	0.922	1.025(0.410-2.562)			
Positive EOT PET/CT (DS 4 or 5)	<0.001	1.057 (1.027-1.087)	<0.001		1.055 (1.025-1.085)

PFS, progression free survival; OS, overall survival; CI, confidence interval; ECOG, Eastern Cooperative Oncology Group; LDH, lactate dehydrogenase; IPI, International prognostic index; EOT-PET, end of treatment PET/CT; DS, Deauville Score.

### Survival Analysis According to the EOT-PET Results

**Figure 3:**
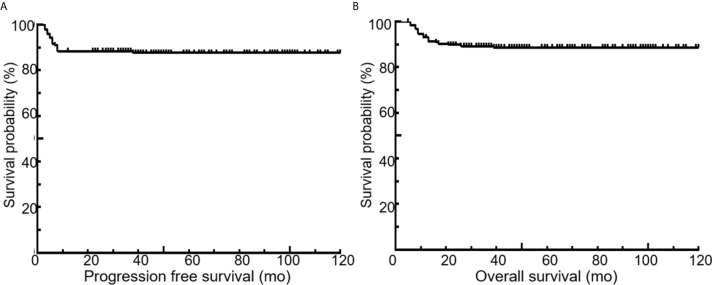
Kaplan-Meier curves for PFS **(A)** and OS **(B)** of the entire patient population. Five-year PFS and OS, 88.3% and 89.3%, respectively.

**Figure 4:**
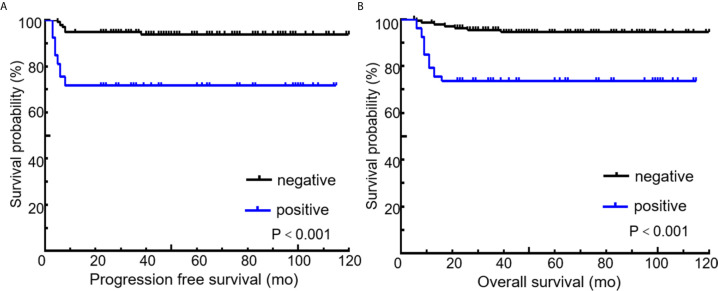
Kaplan-Meier curves for PFS **(A)** and OS **(B)** according to end of treatment PET/CT results using the recommended cutoff value by the Deauville criteria. Deauville score of 1 to 3 means negative and 4 to 5 means positive.

**Figure 5:**
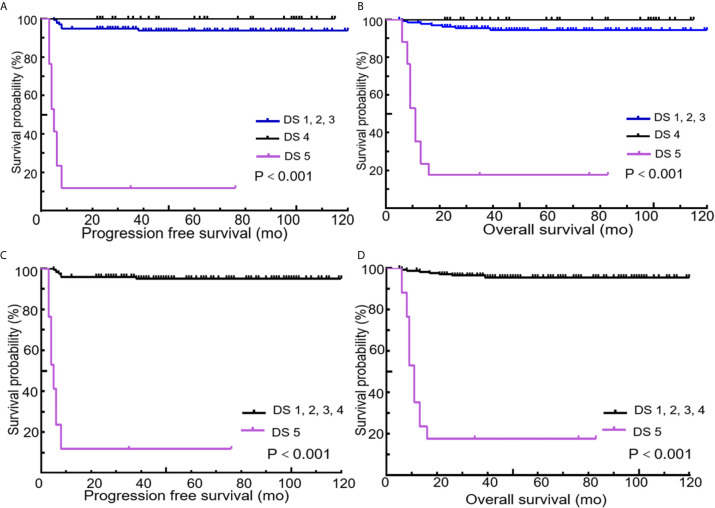
Kaplan-Meier curves of PFS and OS according to end of treatment PET/CT results using different cutoff values of the Deauville score (Cutoff at 1 to 3 vs. 4 vs. 5 or 1 to 4 vs. 5). Cutoff at 1 to 3 vs. 4 vs. 5 for PFS **(A)** and OS **(B)**. Cutoff at 1 to 4 vs. 5 for PFS **(C)** and OS **(D)**.

All 189 patients underwent EOT-PET. 97 patients with baseline PET/CT were all positive showing the presence of at least one hypermetabolic lesion. 136 (72.0%) patients were negative on the EOT- PET scan and 53 (28.0%) patients were positive. 17 (32.1%) patients showed DS 5 and 36 (67.9 %) patients showed DS 4 among the 53 positive patients. During the media follow up period of more than 50 months, the 5-year rates of PFS and OS were 88.3% and 89.3%, respectively (**Figure 3**). Patients with negative EOT-PET showed better outcomes compared with those with positive EOT-PET for both PFS (HR = 5.636, 95%CI = 2.837-13.305, p<0.001) and OS (HR = 5.842, 95%CI = 2.355-14.490, p<0.001) (**Figure 4**). To further evaluate the survival stratified by scores of 4 and 5, we analyzed all the patients in accordance with DS 1 to 3, DS 4 and DS 5 (**Figure 5**). The PFS rates were 94.1%, 100% and 11.8% for DS 1-3, DS 4 and DS 5, respectively (p=0.001). The OS rates were 94.9%, 100% and 17.6% for DS 1-3, DS 4 and DS 5, respectively (p=0.001). Considering almost an identical PFS and OS between DS 1 to 3 and DS 4, We analyzed patients with DS 1 to 4 and DS 5. Patients with DS 5 showed a significantly inferior PFS and OS compared with those with DS 1 to 4 (PFS: 11.8% vs 95.3%, p<0.001; OS: 17.6% vs 95.9%, p<0.001) (**Figure 5**).

### Predictive Values of EOT-PET According to Assessment Method

136 (72%) patients were classified as negative category and 53 (28%) patients as positive if DS 4 and 5 were considered as positive. 172 (91%) patients were classified as negative and 17 (9%) patients as positive if DS 5 was considered as positive. Given that DS 4 and 5 were considered as positive, the sensitivity, specificity, PPV, and NPV of EOT PET for PFS were 65.2%, 77.1%, 28.3% and 94.1%, respectively and for OS were 66.7%, 76.8%, 26.4% and 94.9%, respectively (**Table 3**). Given that 5-DS 5 was considered as positive, the sensitivity, specificity, PPV, NPV of EOT PET for PFS were 65.2%, 98.8%, 88.2% and 95.3%, respectively and for OS were 66.7%, 98.2%, 82.3% and 95.9% (table 3). AUCs of PFS and OS for DS 4 to 5 were 0.71 (95% CI: 0.59-0.83) and 0.72 (95% CI: 0.59-0.84), respectively. AUCs of PFS and OS for DS 5 were 0.82 (95% CI: 0.70-0.94) and 0.82 (95% CI: 0.70-0.95), respectively.

**Table 3:** Sensitivity, specificity, predictive values and AUC for outcomes.

	DS 1,2,3 vs DS 4, 5	DS 1, 2, 3, 4 vs DS 5
PFS		
Sensitivity	65.20%	65.20%
Specificity	77.10%	98.80%
PPV	28.30%	88.20%
NPV	94.10%	95.30%
AUC (95% CI)	0.71 (0.59-0.83)	0.82 (0.70-0.94)
OS		
Sensitivity	66.70%	66.70%
Specificity	76.80%	98.20%
PPV	26.40%	82.30%
NPV	94.90%	95.90%
AUC (95% CI)	0.72 (0.59-0.84)	0.82 (0.70-0.95)

PFS, progression free survival; OS, overall survival; CI, confidence interval; AUC, Area under the curve; PPV, positive predictive value; NPV, negative predictive value.

## Discussion

Currently, DS is the standard method in international guideline of ^18^F-FDG PET/CT examinations in FDG-avid lymphoma (17). Previous studies has demonstrated that BL is a lymphoma with high cell turnover and with high ^18^F-FDG avidity (10, 14, 15, 23, 24). However, there were only initial and controversial evidences in BL of this method, and the potential effect has not been confirmed (13, 15). In this retrospective study of 189 patients with newly diagnosed BL, we found that patients with positive EOT-PET showed inferior outcome compared with those with negative EOT-PET for both PFS and OS, proving that DS is applicable to BL for response evaluation. However, the PPV is too low (28.3% for PFS, 26.4% for OS) despite a high NPV (94.1% for OS and 94.9% for OS). We demonstrated that patients with DS 5 on EOT-PET showed an inferior outcome compared with DS 1 - 4 with a satisfying PPV (88.2% for PFS, 82.3% for OS). Our study suggests that DS of 5 on EOT-PET provides a better prognosis stratification.

DS of 4 or 5 is defined as the sites of previous lesions FDG uptake on EOT- PET is more than the liver in the Deauville Criteria (25), which usually considered as residual lesions (17). However, it would lead to improper judging of the vitality of residual disease for the pitfall of FDG PET/CT lies in the false-positive results. Our study demonstrated a sensitivity of 65.2%, specificity of 77.1%, PPV of 28.3%, NPV of 94.3% with the cutoff of DS 4 to 5. Consequently, the patients of DS 4 to 5 were a heterogeneous group with different prognoses. This may be due to the pathological nature of BL which contains a large number of macrophages and histiocytes and as a result of chemotherapy that causes much inflammatory reaction [6]. Inflammation might cause false positive FDG uptake. FDG accumulates in several types of inflammatory cells such as lymphocytes, neutrophils and macrophages because their glucose metabolism increases (26, 27).

When patients of DS 4 to 5 were considered as positive, the results were similar with previous studies (12, 28), which showed a low PPV. Raid et al. (28) founded the high incidence of tumor necrosis and inflammation after chemotherapy for BL and consequently, the value of PPV for ^18^F-FDG study is low (25%). Carrillo et al. (12) analyzed 32 patients with BL, and founded that the NPV was 100% and the PPV was 20% for ^18^F-FDG PET/CT and all false-positive lesions were DS 4. In the current study, all patients with DS 4 (36 patients) also had good prognosis without death or relapse. Six out of these 36 patients were pathologically confirmed true-false positive which showed a reactive infiltrate of macrophages, particularly foamy cells and a few lymphoid aggregates, but no viable lymphoma cells. It suggested that a significant number of patients with DS 4 on EOT-PET are made up with inflammatory lesions, contributing to false positive results.

In addition, BL is one of the most rapidly growing tumors with short doubling time (29). Biopsy specimens of BL are often necrotic, as the rapidly growing tumor outstrips the available nutrient supply (30). Therefore, the tumor mass shrinks rapidly after short-term, high-intensity chemotherapy, and the necrotic tissue is likely to remain, rather than being quickly removed like tumor cells. On the other hand, high-intensity chemotherapy causes massive death of cancer cells and surrounding tissues, triggering a strong tumor-related inflammatory response (31), which may lead false positive. The other reason is that FDG nonspecific increased uptake may also occur in radiotherapy-related inflammation. Although the finding of this study was barely affected by radiotherapy, it is recommended that EOT-PET scans should be performed three months after radiotherapy according to the published guidelines (32) in order to avoid false-positives caused by post-radiotherapy inflammation. Moreover, a huge shrinkage of the tumor mass may be able to stimulate an unspecific tissue reaction to remove necrotic tumor remnants. Spaepen k et al. (33) showed that chemotherapy followed by inflammatory reactions in the tumor, leading to increased glucose metabolism unrelated to malignant growth by experiments in lymphoma-bearing animals. In mice, tumor infiltration by mononuclear cells peaks at day 10 after the last cycle of chemotherapy and is still above background on day 15 (33). The last reason we can’t ignore may be the effect of rituximab treatment. Previous study (34) demonstrated that rituximab may involve inflammatory changes associated with recruitment of immune cells. However, the results of the current study did not seem to support this hypothesis. About 80% of patients combined the application of rituximab in this study, but the incidence of DS 4 of the patients who received rituximab was higher than those who didn’t receive rituximab (16.7% vs 28.2%). Future prospective studies are required to explore and confirm this clinical issue.

In this study, we changed the threshold to as high as DS 5, the number of false-positive was minimized and the PPV was evidently improved (88.2% for PFS, 82.3% for OS), compared with DS 4 and 5 as being the threshold (28.3% for PFS, 26.4% for OS). In the case of maintaining sensitivity without decreasing, moving the cutoff point to DS 5 for positive EOT-PET resulted in a distinction between EOT-PET negative and positive patients in both PFS and OS with increased specificity, PPV and NPV. Therefore, two clustered prognostic groups were showed on EOT-PET, one with superior prognosis in DS scores of 1 to 4 and one with inferior prognosis in DS score of 5. This finding suggests that a significant proportion of patients would be overtreated and increase the risk of unnecessary treatment-related toxicity if DS 4 to 5 was defined as positivity in clinical trials. Previous researches (12, 28, 35) similar to the current study had confirmed a high rate of tumor necrosis and inflammation after chemotherapy for the BL and consequently, the incidence of true-positive 18F-FDG is low. However, to the best of our knowledge, there is currently no literature to suggest a solution to solve the problem of the low true-positive rates. In the current study, we innovatively moved the cutoff point to DS 5; the PPV was improved evidently. According to our study, DS 4 may stand for complete response. However, inadequate treatment should also be avoided and histopathological confirmation of positive lesions may be necessary before further treatment.

Our study had certain limitations. The primary limitation is its retrospective nature. In addition, this study is also limited by the heterogeneity of patient age (110 children and 79 adults). However, although age has been shown to be a factor of prognostic, there is no need to stratify age when using Lugano criteria to perform response evaluation (17). Finally, the treatment schemes were heterogeneous, which may influence the FDG uptake in the patients with complete response and leads to a false-positive result. Thus, a prospective validation is necessary with a longer follow-up in defining DS 5 for the response assessment in patients of BL with monotherapy. Despite this, the present study represents the first series of BL conducted with different cutoff values to evaluate the usefulness of EOT-PET so far.

In conclusion, EOT-PET results stratified by DS could effectively predict the PFS and OS. The DS 4 may be not the most appropriate cutoff value, which may overestimate the proportion of patients with residual disease. Our study shows that DS 5 may be a better cutoff point at the end of treatment, which helps to determine whether patients have a true risk of recurrence or progression.

## Data Availability Statement

The original contributions presented in the study are included in the article/supplementary material. Further inquiries can be directed to the corresponding authors.

## Ethics Statement

Written informed consent was not obtained from the individual(s), nor the minor(s)’ legal guardian/next of kin, for the publication of any potentially identifiable images or data included in this article.

## Author Contributions

WF and XZ: conception and design, approved the final version of the manuscript on behalf of all authors, administrative/technical/material support, and study supervision. YM, ZX, YW, and XL: acquisition of the data, analysis, and interpretation of the data. YM, ZX, YW, and XL: drafting of the article. All authors: critical revision of the article and reviewed submitted version of the manuscript. All authors contributed to the article and approved the submitted version.

## Conflict of Interest

The authors declare that the research was conducted in the absence of any commercial or financial relationships that could be construed as a potential conflict of interest.
